# Short-term adverse remodeling progression in asymptomatic aortic stenosis

**DOI:** 10.1007/s00330-020-07462-9

**Published:** 2020-11-19

**Authors:** Anvesha Singh, Daniel C. S. Chan, Prathap Kanagala, Kai Hogrefe, Damian J. Kelly, Jeffery P. Khoo, David Sprigings, John P. Greenwood, Ahmed M. S. E. K. Abdelaty, Michael Jerosch-Herold, Leong L. Ng, Gerry P. McCann

**Affiliations:** 1 Department of Cardiovascular Sciences, University of Leicester and Cardiovascular Theme, NIHR Leicester Biomedical Research Centre, Glenfield Hospital, Groby road, Leicester, LE3 9QP UK; 2 Department of Cardiology, Aintree University Hospital, Liverpool, UK; 3 Cardiology Department, Kettering General Hospital Foundation Trust, Rothwell Rd, Kettering, NN16 8UZ UK; 4 Cardiology Department, Royal Derby Hospital, Uttoxeter Rd, Derby, DE22 3NE UK; 5 Cardiology Department, Glenfield Hospital, Groby Road, Leicester, LE3 9QP UK; 6 Northampton General Hospital, Cliftonville, Northampton, NN1 5BD UK; 7 Multidisciplinary Cardiovascular Research Centre & The Division of Biomedical Imaging, Leeds Institute of Cardiovascular & Metabolic Medicine, Leeds University, Leeds, LS2 9JT UK; 8 Cardiology Department, Suez Canal University, Ring road, Ismailia, 41611 Egypt; 9 Brigham and Woman’s Hospital and Harvard Medical School, 75 Francis St, Boston, MA 02115 USA

**Keywords:** Aortic valve stenosis, Magnetic resonance imaging, Fibrosis

## Abstract

**Objectives:**

Aortic stenosis (AS) is characterised by a long and variable asymptomatic course. Our objective was to use cardiovascular magnetic resonance imaging (MRI) to assess progression of adverse remodeling in asymptomatic AS.

**Methods:**

Participants from the PRIMID-AS study, a prospective, multi-centre observational study of asymptomatic patients with moderate to severe AS, who remained asymptomatic at 12 months, were invited to undergo a repeat cardiac MRI.

**Results:**

Forty-three participants with moderate-severe AS (mean age 64.4 ± 14.8 years, 83.4% male, aortic valve area index 0.54 ± 0.15 cm^2^/m^2^) were included. There was small but significant increase in indexed left ventricular (LV) (90.7 ± 22.0 to 94.5 ± 23.1 ml/m^2^, *p* = 0.007) and left atrial volumes (52.9 ± 11.3 to 58.6 ± 13.6 ml/m^2^, *p* < 0.001), with a decrease in systolic (LV ejection fraction 57.9 ± 4.6 to 55.6 ± 4.1%, *p* = 0.001) and diastolic (longitudinal diastolic strain rate 1.06 ± 0.2 to 0.99 ± 0.2 1/s, *p* = 0.026) function, but no overall change in LV mass or mass/volume. Late gadolinium enhancement increased (2.02 to 4.26 g, *p* < 0.001) but markers of diffuse interstitial fibrosis did not change significantly (extracellular volume index 12.9 [11.4, 17.0] ml/m^2^ to 13.3 [11.1, 15.1] ml/m^2^, *p* = 0.689). There was also a significant increase in the levels of NT-proBNP (43.6 [13.45, 137.08] pg/ml to 53.4 [19.14, 202.20] pg/ml, *p* = 0.001).

**Conclusions:**

There is progression in cardiac remodeling with increasing scar burden even in asymptomatic AS. Given the lack of reversibility of LGE post-AVR and its association with long-term mortality post-AVR, this suggests the potential need for earlier intervention, before the accumulation of LGE, to improve the long-term outcomes in AS.

**Key Points:**

*• Current guidelines recommend waiting until symptom onset before valve replacement in severe AS.*

*• MRI showed clear progression in cardiac remodeling over 12 months in asymptomatic patients with AS, with near doubling in LGE.*

*• This highlights the need for potentially earlier intervention or better risk stratification in AS.*

## Introduction

Aortic stenosis (AS) is the commonest valve lesion requiring surgery in the developed world, with increasing prevalence with ageing populations [1]. It is characterised by a long and variable asymptomatic course. The development of myocardial fibrosis is key in the transition from compensated hypertrophy to heart failure, with low-grade inflammation also playing a role [2].

Cardiovascular magnetic resonance imaging (MRI) has provided valuable insights into the remodeling patterns in AS [3]. Several MRI-measured markers have been linked to symptoms, exercise capacity and outcome in AS, including myocardial perfusion reserve (MPR) [4, 5]; surrogate markers of diffuse interstitial fibrosis: T1, extracellular volume fraction (ECV) [6], absolute extracellular volume index (iECV) [7]; and extent of focal fibrosis measured by late gadolinium enhancement (LGE) [8]. A large multi-centre study has confirmed LGE to be independently associated with mortality even after aortic valve replacement (AVR) [9]. Others have shown LGE to be irreversible 1–2 years after AVR [10, 11], suggesting a need for potentially earlier intervention before LGE is established and the need for potentially reversible markers to identify those for earlier intervention.

Studying the changes in remodeling at an earlier stage of disease may provide important insights into the pathophysiology of disease progression [12]. The aims of this study were to establish the extent of progression in adverse remodeling within 12 months in asymptomatic patients with AS.

## Methods

### Subjects

Asymptomatic patients with moderate to severe AS were recruited as part of the multi-centre, prospective ‘PRognostic Importance of MIcrovascular Dysfunction in asymptomatic patients with AS’ (PRIMID-AS) study between April 2012 and October 2013 [5, 13]. Their asymptomatic status was confirmed by the clinical team whose care they were under, as well as by the patients. Inclusion criteria were ages 18 to 85 years, moderate to severe AS (≥ 2 of aortic valve area < 1.5 cm^2^, peak pressure gradient > 36 mmHg, or mean pressure gradient > 25 mmHg), asymptomatic, and ability to perform bicycle exercise test. Exclusion criteria were absolute contraindications to MRI/adenosine/contrast, previous cardiac surgery, left ventricular ejection fraction (LVEF) < 40%, persistent atrial flutter/fibrillation, other severe valve disease, previous heart failure, planned AVR or comorbidity limiting life expectancy, or precluding AVR. Those who remained asymptomatic at 12 months on clinical review and direct questioning were invited for a repeat MRI and blood sampling. The UK national research ethics service approved the study (11/EM/0410) and written informed consent was obtained from all participants.

### Investigations

#### Echocardiography

A trans-thoracic echocardiogram was performed at baseline by an accredited sonographer according to international guidelines [14]. All image analysis was conducted at the core lab by a single physiologist, using an Xcelera (Phillips) workstation.

#### MRI

Patients underwent comprehensive multi-parametric 3T cardiac stress MRI including long- and short-axis cine, pre- and post-contrast T1 mapping, adenosine stress first-pass perfusion imaging, and LGE, at baseline and 12 months, using identical imaging protocol, as previously described [13]. T1 mapping was performed on a single mid-ventricular slice. A full left atrial (LA) and left ventricular (LV) short-axis stack was acquired for volumetric assessment. All image analysis was undertaken at the core lab by a single observer (A.S.), blinded to the patient data. Volumetric, T1, and LGE analyses were performed using *cvi42* version 5 (Circle Cardiovascular Imaging). Papillary muscles were excluded from the myocardial mass. LGE was quantified using > 5SD above the mean signal intensity of normal myocardium [15]. ECV was calculated [16] using haematocrit measured on the same day. iECV (ECV × myocardial volume index) and myocyte volume index ([1-ECV] × myocardial volume index) were calculated [17]. To account for a change of flip angle in the T1 sequence between scans, only ECV-derived measures are shown. Absolute myocardial blood flow (MBF) was calculated using model-independent deconvolution method, using Q-mass version 7.1 (Medis), as previously described [18]. MPR was calculated as the ratio of stress MBF to rest MBF. Diogenes feature tracking software (TomTec Imaging Systems) was used for strain and strain rate analysis [19].

**Fig. 1:**
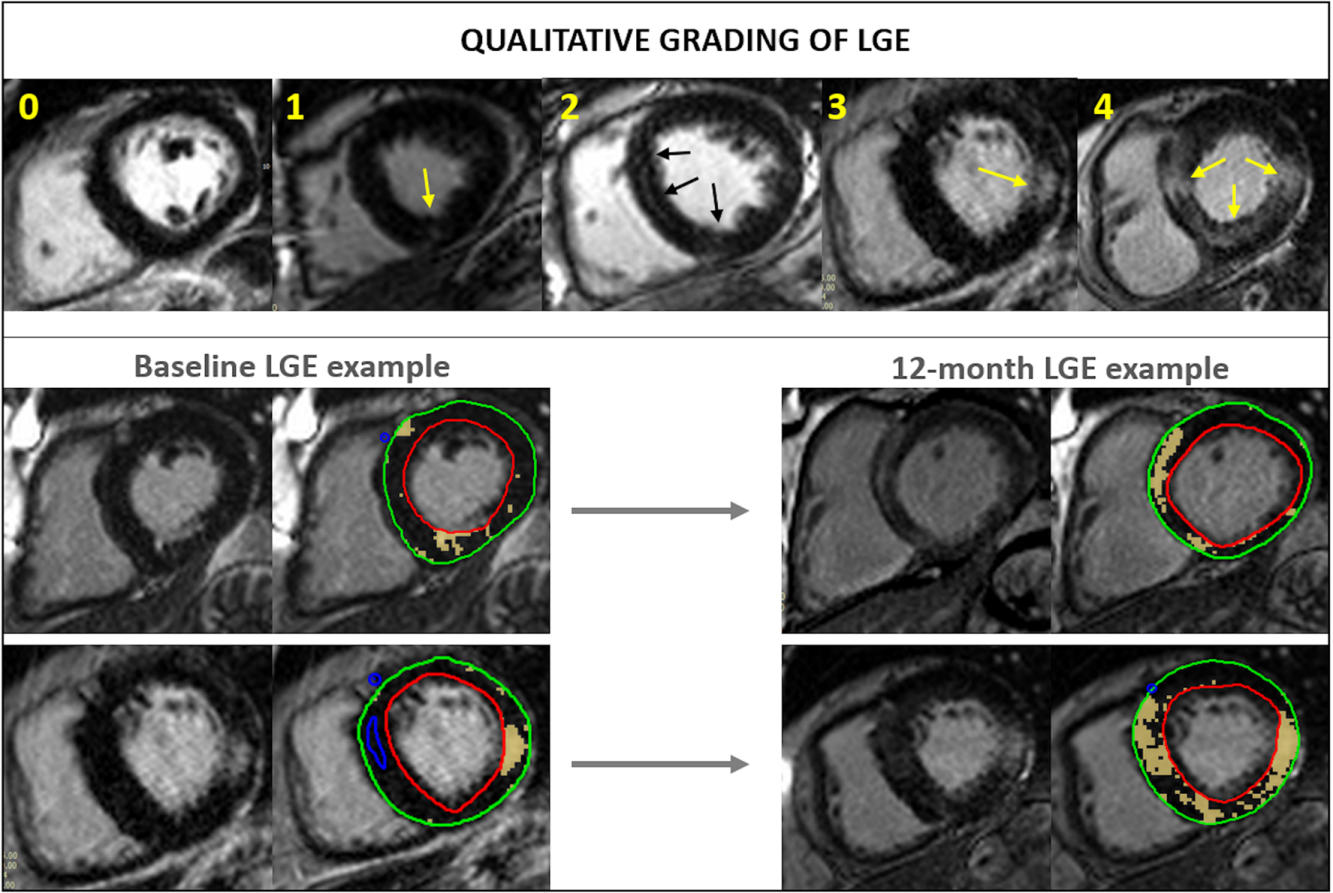
Qualitative non-infarct pattern LGE grading used is demonstrated in the top panel (0 = no enhancement, 1 = insertion point enhancement, 2 = subtle enhancement in 1 region outside insertion point, 3 = bright scar in 1 region/diffuse enhancement in multiple regions, 4 = clear scar in multiple regions). Typical infarct pattern LGE (subendocardial pattern in a coronary artery territory distribution) was also recorded separately. Examples of LGE progression over 12 months shown without and with quantification contours (red region of interest (ROI): endocardial contour; green ROI: epicardial contour; blue ROI: area of normal myocardium defined by the user; yellow ROI: automated areas of LGE detected by software using 5 standard deviation method

Qualitative LGE assessment was performed by 2 assessors (A.S., G.P.M.) and non-infarct pattern LGE was graded as 0 = no enhancement, 1 = mild insertion point enhancement, 2 = subtle enhancement in 1 region outside insertion point, 3 = bright scar in 1 region/diffuse enhancement in multiple regions, and 4 = clear scar in multiple regions (Fig. 1). Typical infarct pattern LGE affecting the subendocardium in a coronary artery territory distribution was also recorded.

#### Plasma

Blood samples were collected in EDTA tubes and centrifuged within 4 h at 2000*g* for 20 min. Plasma was drawn off and stored at − 80 °C. Blinded, single-batch analysis was performed at the end. N-Terminal-pro brain natriuretic peptide (NT-proBNP) was measured using our in-house non-competitive assay that employs the quantitative sandwich enzyme immunoassay technique, and has excellent correlation with the Roche Elecsys assay.

### Statistical analysis

Statistical analysis was performed using SPSS 24.0 software (Statistical Package for the Social Sciences), with *p* < 0.05 considered statistically significant. Normally distributed data are expressed as mean ± standard deviation. Non-parametric data are expressed as median (interquartile range). Continuous variables were compared between baseline and follow-up visit using paired sample *t* tests or the Wilcoxon signed-rank test. McNemar test was used for categorical variables. Linear regression was used to explore the univariate associations of baseline MRI parameters/echocardiographic Doppler data/ NTproBNP levels with absolute change in the LV remodeling parameters: LV end-diastolic volume index (LVEDVI), LV mass index (LVMI), LV mass/volume, LVEF, left atrial volume index (LAVI) and LGE.

## Results

### Baseline data

Forty-three subjects (36 male, age 64.4 ± 14.8 years) were included (Table 1). Concomitant hypertension was present in over half the cohort, with diabetes in 4 patients. Most patients had severe AS with an echo-derived aortic valve area index (AVAI) of 0.60 ± 0.14 cm^2^/m^2^. Bicuspid morphology was present in 40%.

**Table 1:** Baseline demographic and echocardiographic data (*n* = 43)

Parameter	Value
Age, years	64.4 ± 14.8
Male sex, *n* (%)	36 (83.7)
BMI, kg/m^2^	28.1 ± 4.0
Creatinine	86.88 ± 18.0
Hct	0.43 ± 0.03
Hypertension, *n* (%)	24 (55.8)
Diabetes, *n* (%)	4 (9.3)
Statins, *n* (%)	27 (62.8)
ACE/ARB, *n* (%)	20 (46.5)
Beta-blockers, *n* (%)	12 (27.9)
Ca channel blockers, *n* (%)	11 (25.6)
Moderate AS, *n* (%)	13 (30.2)
Severe AS, *n* (%)	30 (69.8)
Bicuspid valve, *n* (%)	17 (39.5)
AV Vmax, m/s	3.78 ± 0.48
MPG, mmHg	33.74 ± 11.30
AVA, cm^2^	1.20 ± 0.33
AVAI, cm^2^/m^2^	0.60 ± 0.14
E/A	0.91 ± 0.31
Septal E/e’	12.8 ± 3.3
Lateral E/e’	9.8 ± 3.5

*BMI* body mass index, *HR* heart rate, *SBP/DBP* systolic/diastolic blood pressure, *Hct* haematocrit, *ACE-I* angiotensin-converting enzyme inhibitor, *ARB* angiotensin II receptor blocker, *AV Vmax* peak aortic jet velocity, *MPG* mean pressure gradient, *AVAI* aortic valve area indexed to BSA, *AS* aortic stenosis, *LGE* late gadolinium enhancement

### Remodeling at 12 months

There was no significant change in patients’ weight, blood pressure, or heart rate between baseline and follow-up. MRI data is shown in Table 2. Paired T1 maps were available in 25 patients (baseline native T1 1114.9 ± 56.7 ms) as the T1 mapping sequence was not available from the vendor during parts of the study. Follow-up LGE imaging was acquired in 42 patients. MRI planimetry AVAI remained unchanged at 12 months. There was a significant increase in indexed LV and LA volumes, with the increase in right ventricular (RV) volumes of borderline statistical significance (*p* = 0.05). These were associated with a small but significant decrease in LV and RV EF, albeit remaining within the normal range. The longitudinal peak diastolic strain rate also decreased significantly. There was a borderline significant increase in LVMI (*p* = 0.058) but no significant change in LV mass/volume.

**Table 2:** MRI and biomarker data at baseline and 12 months

Parameter	Baseline	12 months	*p* value
AVAI, cm^2^/m^2^	0.54 ± 0.15	0.50 ± 0.11	0.133
LVEDVI, ml/m^2^	90.7 ± 22.0	94.5 ± 23.1	0.007
LVESVI, ml/m^2^	38.7 ± 12.3	42.5 ± 13.6	< 0.001
LVEF, %	57.9 ± 4.6	55.6 ± 4.1	0.001
LAVI, ml/m^2^	52.9 ± 11.3	58.6 ± 13.6	< 0.001
RVEDVI, ml/m^2^	89.0 ± 13.3	92.3 ± 13.5	0.050
RVEF, %	56.8 ± 6.2	54.5 ± 4.6	0.006
LVMI, g/m^2^	59.1 ± 15.1	60.6 ± 16.0	0.058
LV mass/volume, g/ml	0.66 ± 0.10	0.64 ± 0.09	0.231
Septal wall thickness, cm	1.42 ± 0.26	1.39 ± 0.26	0.093
Longitudinal PSS, %	− 18.5 ± 2.8	− 18.3 ± 2.4	0.537
Longitudinal PEDSR, 1/s	1.06 ± 0.24	0.99 ± 0.24	0.026
ECV fraction, %	24.2 ± 2.03	24.3 ± 4.66	0.929
Extracellular volume index, ml/m^2^	12.9 (11.4, 17.0)	13.3 (11.1, 15.1)	0.689
Cellular volume index, ml/m^2^	42.0 (37.4, 48.3)	41.5 (36.6, 48.2)	0.265
Qualitative LGE > grade 1, *n* (%)	11 (26)	20 (48)	0.001
0 - Normal	7	3	
1 - Insertion point only	15	10	
2 - Subtle enhancement in 1 region	2	9	
3 - Bright scar in 1 region/diffuse subtle enhancement	7	8	
4 - Scar in multiple regions	2	3	
Infarct pattern	9	9	
LGE, g	2.02 (1.26, 4.57)	4.26 (2.17, 6.85)	< 0.001
LGE % LVmass	2.25 (1.03, 4.10)	4.20 (2.30, 6.40)	< 0.001
Rest MBF, ml/min/g	0.63 (0.54, 0.78)	0.56 (0.44, 0.62)	< 0.001
Stress MBF, ml/min/g	1.34 (1.03, 1.54)	1.08 (0.92, 1.34)	0.002
MPR	2.01 ± 0.58	2.17 ± 0.76	0.243
NT-proBNP, pg/mL	43.6 (13.45, 137.08)	53.4 (19.14, 202.20)	0.001

*BMI* body mass index, *HR* heart rate, *SBP/DBP* systolic/diastolic blood pressure, *Hct* haematocrit, *ACE-I* angiotensin-converting enzyme inhibitor, *ARB* angiotensin II receptor blocker, *AV Vmax* peak aortic jet velocity, *MPG* mean pressure gradient, *AVAI* aortic valve area indexed to BSA, *AS* aortic stenosis, *LGE* late gadolinium enhancement, *LVEDVI* left ventricular end-diastolic volume index (BSA), *LVESVI* left ventricular end-systolic volume index, *LVSVI* left ventricular stroke volume index, *LVEF* left ventricular ejection fraction, *LVMI* left ventricular mass index, *LAVI* left atrial volume index, *RVEDVI* right ventricular end-diastolic volume index, *PSS* peak systolic strain, *PEDSR* peak early diastolic strain rate, *MPR* myocardial perfusion reserve, *MBF* myocardial blood flow, *LGE* late gadolinium enhancement, *ECV* extracellular volume. Paired T1 mapping available in *n* = 25, paired LGE analysis possible in *n* = 42, and myocardial blood flow data available in *n* = 41

### Change in measures of fibrosis

**Fig. 2:**
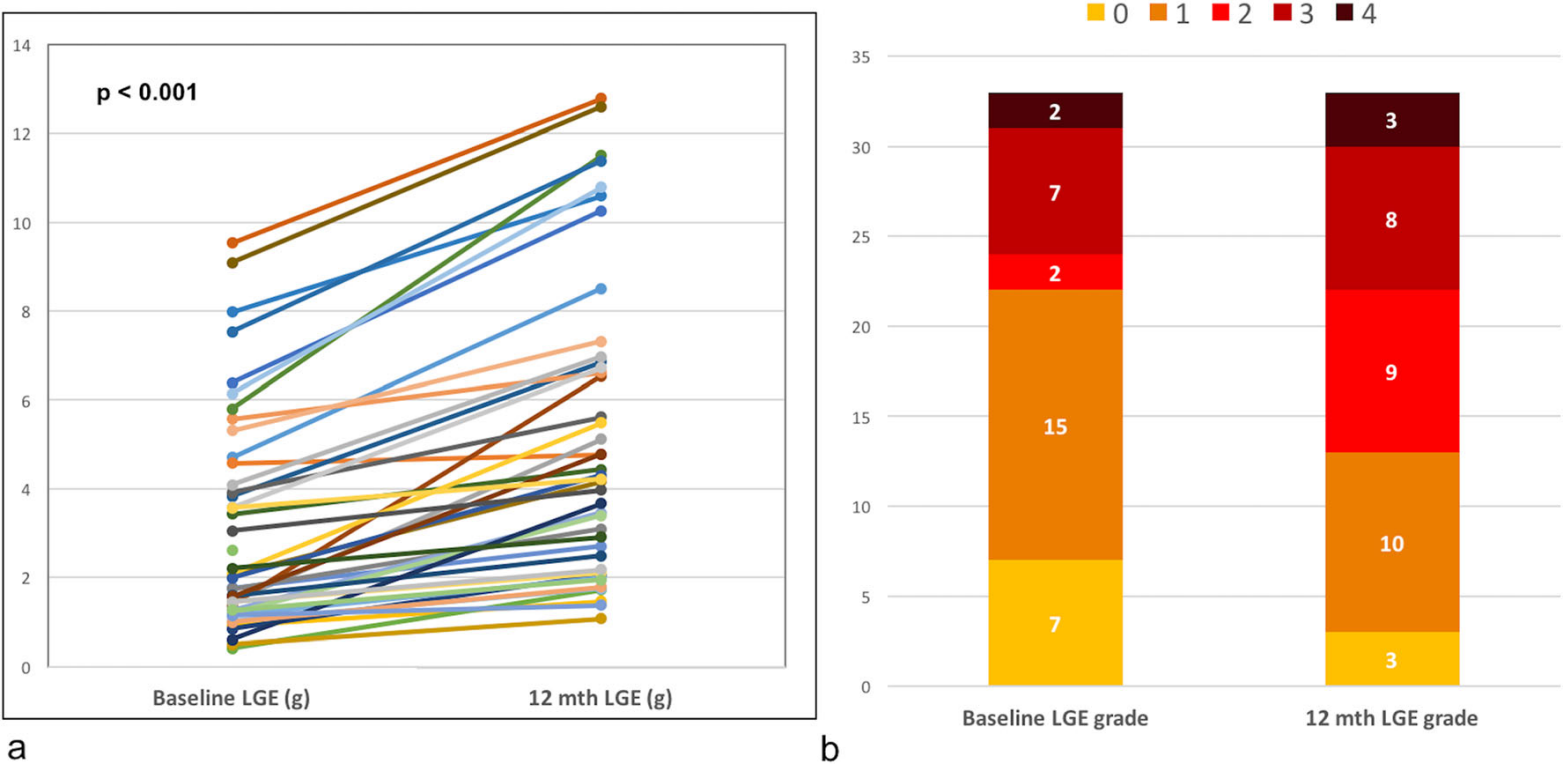
**a** Change in LGE from baseline to 12 months. **b** Qualitative grading of LGE at baseline and 12 months (excluding infarct/no follow-up LGE)

Significant non-infarct pattern LGE (> grade 1 on qualitative analysis) was present in 11 patients at baseline, and 20 at follow-up, with subendocardial infarction in an additional 9 patients at both visits and no new infarctions at follow-up. Excluding those with infarct pattern LGE and with missing follow-up LGE imaging (*n* = 1), the qualitative grade of LGE increased in 39% (13 out of 33 patients, and in 11 out of 33 to grade 2 or higher) (Figs. 1 and 2), remained unchanged in 52% (*n* = 17), and decreased by one grade in 3 patients (on blinded analysis). The total amount of LGE(g) doubled over 12 months. There was no significant change in the measures of diffuse interstitial fibrosis: ECV or iECV. There was a decrease in stress and rest MBF, but no change in the MPR.

### Plasma biomarker

There was a significant increase in levels of NT-proBNP at 12 months (Table 2). The baseline level of NT-proBNP was significantly correlated with baseline LAVI (*r* = 0.35, *p* = 0.021).

### Associations with change in remodeling parameters

On univariate analysis, baseline LV mass/volume was positively associated with change in LVEDVI, and negatively associated with change in LV mass/volume (Table 3). For change in LVEF, there were significant negative associations with baseline measures of AS severity and LVEF. Baseline LGE, LAVI, native T1, and NT-proBNP associated positively with change in LGE.

**Table 3:** Significant univariate associations of baseline parameters with absolute change in LV remodeling parameters on linear regression analysis

Baseline MRI parameter	Estimate (95% CI)	*p* value
Change in LVEDVI		
Baseline LV mass/volume	40.1 (15.1, 65.2)	0.002
Change in LV mass/volume		
Baseline LV mass/volume	− 0.32 (− 0.52, − 0.12)	0.002
Baseline LAVI	0.002 (0.000, 0.004)	0.020
Change in LVEF		
Baseline AV Vmax	− 3.20 (− 5.76, − 0.64)	0.015
Baseline PPG	− 0.10 (− 0.18, − 0.02)	0.018
Baseline MPG	− 0.12 (− 0.23, − 0.01)	0.033
Baseline LVEF	− 0.52 (− 0.76, − 0.29)	< 0.001
Change in LGE (g)		
Baseline LGE (g)	0.28 (0.12, 0.45)	0.001
Baseline LAVI	0.05 (0.01, 0.08)	0.022
Baseline native T1	0.01 (0.00, 0.02)	0.006
Baseline NT-proBNP	0.004 (0.001, 0.007)	0.009

*BMI* body mass index, *HR* heart rate, *SBP/DBP* systolic/diastolic blood pressure, *Hct* haematocrit, *ACE-I* angiotensin-converting enzyme inhibitor, *ARB* angiotensin II receptor blocker, *AV Vmax* peak aortic jet velocity, *MPG* mean pressure gradient, *AVAI* aortic valve area indexed to BSA, *AS* aortic stenosis, *LGE* late gadolinium enhancement, *LVEDVI* left ventricular end-diastolic volume index (BSA), *LVESVI* left ventricular end-systolic volume index, *LVSVI* left ventricular stroke volume index, *LVEF* left ventricular ejection fraction, *LVMI* left ventricular mass index, *LAVI* left atrial volume index, *RVEDVI* right ventricular end-diastolic volume index, *PSS* peak systolic strain, *PEDSR* peak early diastolic strain rate, *MPR* myocardial perfusion reserve, *MBF* myocardial blood flow, *LGE* late gadolinium enhancement, *ECV* extracellular volume

## Discussion

In this study, we performed paired MRI and biomarker analysis at baseline and 12 months, in a cohort of patients with significant but asymptomatic AS. We show clear progression in adverse remodeling, with increase in NT-proBNP, despite patients remaining asymptomatic.

### Changes in remodeling

There was a significant increase in ventricular and LA volumes, with a small decrease in function. There is only one other MRI study assessing cardiac remodeling progression in asymptomatic AS, which showed important differences compared to ours [11]. Their LVMI and wall thickness increased, whilst LVEDVI decreased, with no change in LVEF. However, their cohort of 61 included 26 patients with mild AS, and only 14 with severe AS, compared to ours with majority being severe (30/43). This suggests that at an earlier stage of disease, patients develop more concentric remodeling, whilst ours, at a later stage of disease, switched to more eccentric remodeling, with increase in volumes and a non-significant reduction in wall thickness. Increased concentric remodeling (LV mass/volume) at baseline was associated with greater degree of LV dilatation at 12 months, and negatively associated with change in mass/volume, also suggesting more eccentric remodeling with continued pressure overload caused by AS.

Both systolic and diastolic function decreased, and NT-proBNP levels increased, confirming disease progression, despite no significant change in AVA measured on MRI, suggesting progressive cardiac decompensation under chronic pressure overload [20]. It is possible that those with more marked progression in AS were intervened on earlier, and therefore, not included in this cohort. Reduced diastolic function is associated with poor prognosis post-AVR [21], and EF < 60% is associated with disease progression and worse prognosis [22, 23].

### Late gadolinium enhancement

There was marked increase in focal fibrosis. Significant non-infarct pattern LGE (> grade 1) was present in 36% of all patients at baseline and increased to 57% at 12 months. A recent meta-analysis showed LGE to be present in 49.6% of patients with AS [24]. LGE is a poor prognostic marker even after AVR [24, 25] and irreversible once established [10]. However, there was no significant change in ECV, a surrogate of diffuse fibrosis. This may partly be due to T1 mapping only being measured on a single mid-ventricular slice, compared to the full LV being covered for LGE analysis, and the non-ischaemic fibrosis often tends to affect the basal slices in early disease. In Everett’s study [11], there was also significant increase in LGE of 1.6 g/year from a similar baseline value of 2.5 g at baseline, but no change in ECV. Their iECV did increase, due to a corresponding increase in LV mass, which is incorporated in its calculation.

Baseline LGE was positively associated with change in LGE, which is similar to the previous finding of LGE progressing fastest in those with more LGE at baseline [11]. Coronary disease progression is unlikely to be responsible, as there were no new infarcts noted at follow-up. In addition, baseline LAVI and NT-proBNP were also positively correlated with progressive focal fibrosis, again supporting the role of chronic pressure overload caused by AS leading to progressive cardiac decompensation.

### Myocardial blood flow

Both rest and stress MBF decreased, but there was no change in MPR. MBF is quantified per gram of myocardium, and therefore, this most likely represents an increase in non-metabolically active fibrotic burden within the myocardium. In this asymptomatic cohort, with exclusion of those who develop symptoms, perfusion falls but remains balanced. Combined with the findings of MPR being a predictor of symptom onset in the original PRIMID study, this suggests that a separation of the rates of decline in rest and stress MBF may be a key step in symptom onset in AS. This hypothesis will of course need to be tested in a separate and larger cohort.

### Clinical implications

Our data highlights the potential need for earlier intervention or development of anti-fibrotic therapy to optimise long-term outcomes in AS. Recent data suggests the non-inferiority of TAVR in lower risk (but still symptomatic severe) AS [26, 27]. The optimal timing of AVR may be even earlier, in the pre-symptomatic stage, prior to establishment of irreversible remodeling. The EVOLVED (NCT03094143), EARLY TAVR (NCT03042104) and EASY-AS (NCT04204915) trials are evaluating such a strategy.

### Limitations

These patients were a subset of those who remained asymptomatic and were not referred for surgery, and comprise a relatively small cohort, although poorly studied previously. T1 mapping data was not available in all patients, and only measured on a single mid-ventricular slice, whilst LGE was measured using a full short-axis stack. Lack of repeat echocardiography for assessment of AS severity at follow-up is another limitation. However, planimetry AVA on CMR has been shown to be a reliable and reproducible technique, with close agreement with AVA on transoesophageal imaging and AS severity on cardiac catheterisation [28–30].

## Conclusions

Asymptomatic patients with moderate to severe AS demonstrate unequivocal progression in adverse cardiac remodeling within 12 months, with a significant increase in focal myocardial fibrosis. Further studies are required to determine whether earlier intervention in asymptomatic AS is associated with improved long-term outcomes.

## Abbreviations

AS: Aortic stenosis
AVA(I): Aortic valve area (index)
AVR: Aortic valve replacement
ECV: Extracellular volume
iECV: Absolute extracellular volume index
L/RVEDVI: Left/right ventricular end-diastolic volume index
L/RVEF: Left/right ventricle ejection fraction
LA (VI): Left atrial (volume index)
LGE: Late gadolinium enhancement
LVMI: Left ventricular mass index
MBF: Myocardial blood flow
MPR: Myocardial perfusion reserve
MRI: Magnetic resonance imaging
